# Research progress on the therapeutic effects of polysaccharides on non-alcoholic fatty liver diseases

**DOI:** 10.3389/fnut.2023.1107551

**Published:** 2023-03-10

**Authors:** Yu-long Hu, Qiaoli Ma, Xiaoqiang Dong, Yuanfang Kong, Juntao Cai, Jieming Li, Chunhong Dong

**Affiliations:** ^1^Academy of Chinese Medical Science, Henan University of Chinese Medicine, Zhengzhou, China; ^2^Henan Polysaccharide Research Center, Zhengzhou, China; ^3^Henan Key Laboratory of Chinese Medicine for Polysaccharides and Drugs Research, Zhengzhou, China; ^4^College of Pharmacy, Henan University of Chinese Medicine, Zhengzhou, China

**Keywords:** polysaccharides, NAFLD, NASH, therapy, liver diseases

## Abstract

Non-alcoholic fatty liver disease (NAFLD) has become the most common chronic liver disease and is a leading cause of cirrhosis and hepatocellular carcinoma. Due to its complex pathophysiology, there is currently no approved therapy. Polysaccharide, a kind of natural product, possesses a wide range of pharmacological activities. Numerous preclinical studies have confirmed that polysaccharides could interfere with the occurrence and development of NAFLD at multiple interrelated levels, such as improvement of glucose and lipid metabolism, antioxidation, anti-inflammation, and regulation of gut-liver axis, thus showing great potential as novel anti-NAFLD drugs. In this paper, we reviewed the polysaccharides with anti-NAFLD effect in recent years, and also systematically analyzed their possible pharmacological mechanisms.

## Introduction

Non-alcohol fatty liver disease (NAFLD) has become the most prevalent chronic liver disease worldwide, with a global morbidity of more than 32.4%, thus representing an enormous health care burden (1). NAFLD encompasses a wide spectrum of conditions, ranging from simple hepatic steatosis to non-alcoholic steatohepatitis (NASH) with or without fibrosis, which may eventually evolve to cirrhosis or hepatocellular carcinoma (2). The NAFLD pathophysiology is complex, and is not yet fully understood. The current, and most accepted, theory explaining the pathogenesis of NAFLD is the “multiple-hit” hypothesis, which suggests that the progression of NAFLD is a result of a multitude of ‘hits,’ involving hyperlipidemia, insulin resistance (IR), mitochondrial dysfunction, inflammatory cytokines/adipokines, endoplasmic reticulum (ER) stress, dysregulation of intestinal microflora, oxidative stress and environmental and dietary factors (3). Fortunately, Dietary and lifestyle adjustments can effectively control NAFLD, but when these approaches do not work, pharmaceutical intervention is necessary. However, there are still no approved drugs for NAFLD (4, 5).

Natural products contribute greatly to drug discovery and development, and today up to 100 active ingredients derived from plants are used as medicines in clinic. As a major part of natural resources, polysaccharides are captivating growing interest due to their unique advantages, such as low toxicity, high safety, multi-target and multi-pathway. Importantly, polysaccharides have a wide range of pharmacological activities such as anti-inflammatory, antioxidant, anti-fibrotic, immunomodulatory, anti-tumor effects, and so on (6–9). In terms of NAFLD, some progress has been made recently in the study of anti-NAFLD polysaccharides, and numerous preclinical studies have confirmed that polysaccharides could interfere with the occurrence and development of NAFLD by targeting multiple mechanisms. Accordingly, in present review, we systematically summarized the potential anti-NAFLD polysaccharides and their possible pharmacological mechanisms, with the hope to provide a reference for the development of novel carbohydrate drugs against NAFLD.

## Therapeutic effects of polysaccharides on NAFLD

### Targeting glucose and lipid metabolism

The defining characteristic of NAFLD is an excessive buildup of triglycerides (TG) in hepatocytes. Accumulation of TG arises from abnormal lipid metabolism. More specifically, either enhanced fat synthesis and import, or reduced fat consumption and exportation, or a combination of both processes contribute to the lipid accumulation (10). Inhibition of free fatty acids (FFA) influx or *de novo* lipogenesis is conductive to the reduction of TG deposition, while enhancing fatty acid oxidation or export is favorable to lipid removal. Therefore, targeting these metabolic pathways may ameliorate NAFLD. Moreover, IR is always implicated in pathogenesis of NAFLD (11). Considering the potent action of insulin to suppress adipose tissue lipolysis, therefore, mitigating insulin resistance also represents a promising therapeutic strategy.

A polysaccharide from jackfruit pulp (JFP-Ps) was found to improve liver function in high-fat diets (HFD) -induced mice by modulating the expression of genes involved in lipid metabolism, such as peroxisome proliferator activated receptor alpha (*PPARα*), hormonesensitive lipase (*HSL*), carnitine palmitoyltransferase 1A (*CPT1*), lipoprotein lipase (*LPL*), acetyl-CoA carboxylase alpha (*ACC*), fatty acid synthase (*FAS*) and sterol regulatory element binding transcription factor (*SREBP-1c*) (12). The activation of PPAR and AMP-activated protein kinase (AMPK) signaling pathways may be responsible for its therapeutic effect. Wang et al. (13) isolated an acidic heteropolysaccharide from walnut green husk (WGHP), and found that WGHP was able to alleviate NAFLD by improving glucose and lipid metabolism. *Cichorium intybus L.* polysaccharide (CP) was reported to be beneficial for the treatment of metabolic dysfunctions. In NAFLD mice model, Wu et al. (14) found that CP reduced lipid contents through activating AMPK cascade. Another study showed that CP reduced the degree of steatosis by increasing the levels of *L*-palmitoylcarnitine and hexadecanoyl-CoA (15), which played an essential role in the fatty acid *β*-oxidation. Additionally, CP can also inhibit *de novo* lipogenesis by improving the expression of X-box binding protein 1 (*Xbp1*), insulin-induced gene 2 (*Insig2*), and cystathionine-γ-lyase (*Cth*). Interestingly, a similar therapeutic effect was observed in NAFLD zebrafish model. Li et al. (16) found CP could also exert lipid-lowering effect through enhancing fatty acid *β*-oxidation and inhibiting lipogenesis.

A polysaccharide from *Stropharia rugoso-annulata* (ASRP) was an acetylated polysaccharide (17). Studies showed that ASRP could reduce fat synthesis and increase fatty acids *β*-oxidation by activating AMPK/SREBP-1c signaling pathway. In another study, *Gracilaria lemaneiformis* polysaccharide (GLP), a sulfated polysaccharide, was found to dramatically decrease serum total cholesterol (TC), TG and FFA levels in high-fat diet mice and promote the conversion of cholesterol to bile acids, thus improving lipid metabolism (18). *Sargassum fusiforme* polysaccharide (SFPS) was also a sulfated polysaccharide. He et al. (19) found that the lipid-lowering effect of SFPS was achieved by decreasing lipogenic genes (*Srebp, Fas*) expression and increasing the expression of lipolytic genes (*PPARɑ*, *CPT1*). Interestingly，another sulfated polysaccharide from *Enteromorpha prolifera* (EP) was able to regulate cholesterol metabolism. Ren et al. (20) found EP clearly decreased the expression of SREBP-2 and 3-hydroxy-3-methylglutaryl-CoA reductase (HMGCR), which were closely associated with the decreased hepatic cholesterol biosynthesis. Additionally, hydrogen sulfide (H_2_S) has been found to possess many physiological effects. In another study, Ren et al. (21) reported that EP could increase H_2_S production in HFD-induced mice, which may account for the reduced serum TG. *Lycium barbarum* polysaccharide (LBP) was reported to have hepatoprotective efficacy. One study in NASH rat model showed that LBP treatment ameliorated obesity, IR and lipid accumulation, thus counteracting HFD-induced NASH (22). Another study in HFD-induced NAFLD model showed that LBP effectively decreased hepatic TG accumulation, and its regulatory effect was related to the activation of sirtuin 1 (SIRT1)/liver kinase B1 (LKB1)/AMPK pathway (23).

Yan et al. (24) found that *Ginkgo biloba* leaf polysaccharide (GBLP) could play a certain protective role against NAFLD by decreasing hepatic fat accumulation, improving liver function, and ameliorating insulin resistance. Another study showed that *Radix hedysari* polysaccharides (RHP) was able to improve lipid metabolism disorders, decrease hepatic lipid content by regulating the genes involved in lipid metabolism including *PPARα* and *SREBP-1c* (25). A polysaccharide from *Poria cocos* (PCP) was found to improve lipid metabolism in NAFLD mice model (26). Not only did PCP increase liver lipid transportation to the blood by enhancing the expression of genes involved in lipid transportation, such as solute carrier family 27 member 4 (*Scl27a4*), apolipoprotein C2 (*Apoc2*), and *CD36* (a fatty acid translocase), but it could also promote glucose metabolism through enhancing glucose oxidative utilization.

**Figure 1:**
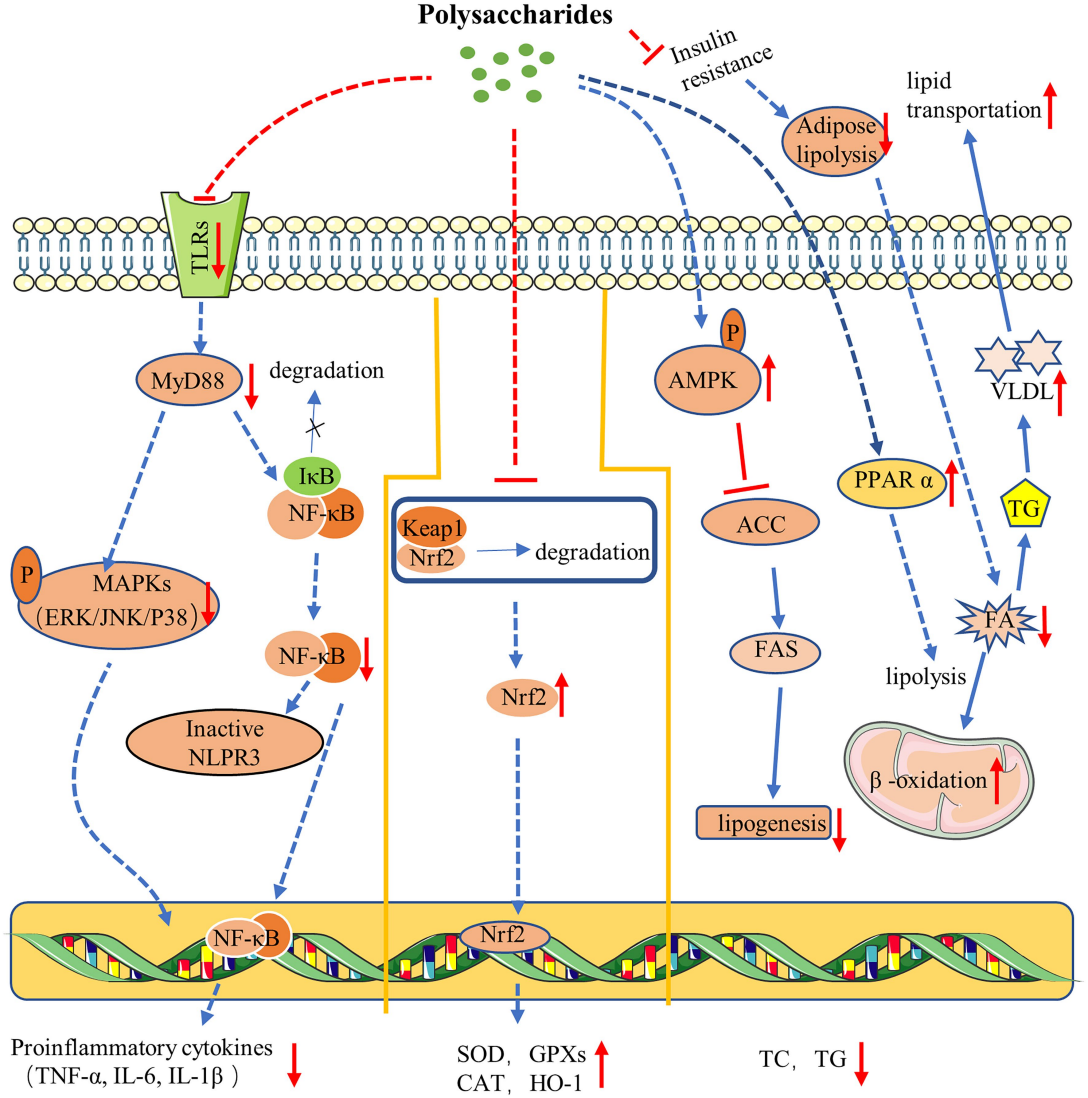
Mechanism of polysaccharides action on NAFLD. Polysaccharides ameliorate NAFLD *via* anti-inflammation **(left)**. Polysaccharides improve NAFLD *via* antioxidation **(middle)**. Polysaccharides exert therapy effect on NAFLD *via* regulating glucose and lipid metabolism **(right)**.

Taken together, polysaccharide exhibited remarkable benefits on glucose and lipid metabolism in NAFLD (Figure 1). Polysaccharide could exert therapeutic effect *via* stimulating hepatic lipolysis, increasing fatty acid oxidation, or inhibiting lipogenesis, which are closely associated with the activation of PPAR and AMPK-related signaling pathways.

### Targeting oxidative stress in NAFLD

Oxidative stress can be defined as overproduction of reactive oxygen species (ROS) and/or a decrease or deficiency in antioxidants. Increasing evidence indicates that oxidative stress plays a central role in NAFLD development (27). Mitochondria are the primary source of ROS and excessive accumulation of ROS may lead to oxidative stress, mitochondria damage, and even cell death. In addition, ROS can also oxidize plasma membranes leading to destruction of biological membranes and production of lipid peroxidation products. Malondialdehyde (MDA) is one of the lipid peroxidation products, which is always regarded as a marker of oxidative damage. Nuclear factor E2-related factor 2 (Nrf2) is a key redox balance regulator and when oxidative stress occurs (28), it will initiate antioxidant defense response by elevating the expression of key antioxidant enzymes including superoxide dismutase (SOD), glutathione peroxidase (GSH-Px) and catalase (CAT), to maintain the redox equilibrium. Therefore, agents capable of improving mitochondrial function and ameliorating oxidative stress may prove useful for NAFLD. Indeed, anti-oxidative therapy in animal studies have already shown a promising therapeutic effect. Hence, targeting oxidative stress may represent a valuable therapeutic strategy.

In a NASH mouse model, LBP significantly reduced MDA and nitrotyrosine while increasing CAT and GSH-Px (29). Furthermore, LBP was also shown to downregulate the expression of cytochrome P450 family 2 subfamily E member 1 (*CYP2E1*), which is a key enzyme responsible for the production of ROS in the liver. Another study also confirmed its beneficial effect. Zhang et al. (30) found that in a NAFLD cell model, LBP clearly decreased lipid accumulation and oxidative stress, as evidenced by the reduction of TG, ALT and AST and by the increase of SOD, CAT and GSH-Px. Interestingly, LBP could also promote mitochondrial biogenesis through activating peroxisome proliferator-activated receptor γ coactivator 1 (PGC-1α)/nuclear factor erythroid 2-like 1 (NRF1) signaling pathway (31).

A polysaccharide from S*agittaria sagittifolia* (SSP) was observed to improve oxidative stress in the HFD-induced NAFLD mouse by enhancing Nrf2 cascade to reduce MDA content, and increase SOD and GSH activity (32). Another polysaccharide ACP isolated from *Malpighia emarginata DC.* was also found to promote the Nrf2 activation, as reflected by significantly reduced MDA, and elevation of SOD, and CAT contents. Moreover, ACP could mitigate mitochondrial dysfunction by decreasing uncoupling protein 2 (UCP2) expression, and increasing mitochondrial complex I, IV, and V activity (33). *Codonopsis lanceolate* polysaccharide (CLPS) was able to decrease MDA level, increase the GSH content, and enhance the expression of antioxidant enzymes such as SOD and CAT, showing a significant antioxidant effect. This protective effect was closely associated with the activation of Nrf2 signaling pathway (34). *Ganoderma lucidum* polysaccharide (FYGL) was a hyperbranched proteoglycan consisting of polysaccharides (77%) and protein (17%). Studies indicated that FYGL could ameliorate ROS and MDA, inhibit lactate dehydrogenase release, elevate SOD content, and enhance total antioxidant capacity (35). Polysaccharide CPP-2 was obtained from *Cyclocarya paliurus*, exhibiting remarkable antioxidant capacity. It was reported that CPP-2 could promote the activities of enzymic and non-enzymic antioxidants involving SOD, CAT and GSH-PX while decreasing the contents of MDA and lipid peroxide (36).

In all, a series of studies have tested the effectiveness of polysaccharides as antioxidants in the treatment of NAFLD (Figure 1). Polysaccharides exert antioxidant effect through promoting the nuclear translocation of Nrf2 protein, which subsequently trigger the expression of numerous downstream antioxidant enzymes, leading to an enhanced cellular antioxidant capacity. What is more, polysaccharide can also improve mitochondrial function through diverse pathways to protect cell from oxidative damage.

### Targeting inflammation in NAFLD

The transition from steatosis to NASH is mainly characterized by the occurrence of inflammation (37). Cytokines and inflammatory cells play a major role in the inflammatory processes. Kupffer cells (KCs) are the tissue-resident macrophages, and serve as the first line of protection for the liver against potential damage (38). Upon abnormal activation, however, KCs will secrete more cytokines including tumor necrosis factor-alpha (TNF-α), interleukin-6 (IL-6) and interleukin-1β (IL-1β), and further aggravate inflammatory infiltration leading to tissue injury. Among these cytokines, TNF-α and IL-6 are critically involved in the pathophysiology of NAFLD. Therefore, reducing the levels of these proinflammatory factors by regulating inflammation process appears as a possible therapeutic approach against NAFLD.

A polysaccharide from *Gynostemma pentaphyllum* (GP) could exert anti-inflammatory effects by regulating the Toll-like receptor 2 (TLR2)/NOD-like receptor protein 3 (NLRP3) signaling pathway (39). In a NASH mice model, GP treatment significantly inhibited the expression of TLRs genes (*TLR1*, *TLR2,* and *TLR4*), reduced the levels of NLRP3, and decreased the expression of pro-inflammatory genes (*IL-1β*, *IL-18rap,* and *TNF-α*). Zhong et al. (40) found that the polysaccharide mAPS from *Astragalus mongholicus* could reduce HFD-induced high expression of TNF-α, TLR4, NLRP3 and phosphorylated- nuclear factor-κB (NF-κB) by downregulating the TLR4/NF-κB signaling pathway. Fucoidan, a sulfated polysaccharide extracted from marine brown algae, was observed to ameliorate hepatic inflammatory status by reducing the hepatic mRNA expressions of *TNF-α* and *IL-1β* (41). TFCP was a kind of crude polysaccharides derived from *Tremella fuciformis*. A recent study by Zhou et al. (42) found that TFCP could ameliorate inflammation in NAFLD mice *via* reducing the expressions of inflammation-associated genes (*IL-1β*, *TLR4*, *TNF-α*, and *IL-6*) and enhancing the HNF4α expression. Li et at (15) found that Chicory polysaccharide (CP) could improve inflammation in NAFLD rats by downregulating the expression of interferon regulatory factor 1 (*Ifr1*), which played a key role in hepatitis.

*Lycium barbarum* polysaccharide (LBP) has been shown to attenuate inflammation. One study showed that LBP treatment significantly decreased pro-inflammatory mediators (IL-6, and TNF-α) expressions, reduced NF-κB activity, and lowered the levels of NLRP3/6, indicating that the action of LBP was implicated in the regulation of NF-κB and NLRP3/6 pathways (43). Another study also confirmed its anti-inflammatory effect. Xiao et at (22) reported that LBP was able to reduce the levels of proinflammatory markers (TNF-α, IL-1β, and COX-2) by decreasing NF-κB activity. An acetylated *Stropharia rugoso-annulata* polysaccharide (ASRP) was found to exert anti-inflammatory effect on NAFLD mice model *via* regulating JNK1/AP-1 signaling pathway. Li et al. (17) reported that ASRP treatment significantly decreased the levels of pro-inflammatory mediators (TNF-α, IL-1β, and IL-6) while increasing the content of anti-inflammatory mediators (IL-10). Additionally, ASRP also decreased p-JNK1, p-c-Jun and p-c-Fos expression.

Collectively, TLRs play an essential role in liver inflammation, and activated TLRs activate the cells and promote the release of proinflammatory cytokines that facilitate the progression of NAFLD, while the polysaccharides could interact with TLRs, and further regulate TLRs-mediated NF-κB and MAPK cascades, thus inhibiting the expression of proinflammatory cytokines and ameliorating inflammation (Figure 1).

### Targeting gut-liver axis

The gut–liver axis represents a complex interplay between gut microbiota, intestinal barrier and liver, and its homeostasis plays a critical role in human health (44–46). The disruption of gut-liver axis, however, often contributes to the pathogenesis of liver diseases. For example, NALFD is the most common chronic liver disease, and diet, drug or other environmental factors may lead to flora disequilibrium or intestinal barrier damage, favoring the occurrence of lipid metabolic dysfunction, oxidative stress, low-grade inflammation, thereby contributing to the onset and progression of this disease. Therefore, prebiotics and probiotics capable of modulating gut microbiota and maintaining intestinal barrier integrity may be promising therapeutic agents for NAFLD.

Polysaccharides could improve NAFLD through modulating the ecological balance of gut microbiota. Hong et al. (47) evaluated the therapeutic effect of *Astragalus* polysaccharides (APS) on HFD-induced NAFLD mice. Studies showed that APS significantly increased the abundance and uniformity of intestinal bacteria. Of note, the richness of *D. vulgaris* from *Desulfovibrio* genus was also improved, which showed a potent anti-NAFLD effect by regulating lipid metabolism genes. Similarly, Wang et al. (48) used HFD-induced NAFLD mice model to investigate the effect and mechanism of *Ophiopogon* polysaccharide (MDG-1). Results indicated that MDG-1 could restore gut microbiota balance and improve relative abundance of beneficial bacteria, thus ameliorating hepatic steatosis.

Polysaccharides could improve NAFLD through protecting the intestinal barrier. Ye et al. (49) evaluated the therapeutic effect of *Poria cocos* polysaccharides (PCP) on HFD-induced NASH mice. It was found that PCP reduced pyroptosis-driven gut vascular barrier disruption and entry of endotoxin into the circulation caused by a high-fat diet, thereby ameliorating the inflammation. In addition, another study by Zhang et al. (50) found that *Artemisia sphaerocephala Krasch* polysaccharide (ASKP) could exert beneficial effect on high fructose-induced NAFLD mice by increasing the relative abundance of *Akkermansia*, which was responsible for the reduced intestinal permeability and LPS leakage.

Polysaccharides could improve NAFLD through regulating the production of short-chain fatty acids (SCFAs). An water insoluble polysaccharide from *Poria cocos* (WIP) was found to significantly increase the relative abundance of butyrate-producing bacteria in NAFLD mice (51), while butyrate could enhance the expression of mucosal integrity proteins (Muc-5) and tight junction proteins in the ileum, to maintain the integrity of the intestinal barrier and prevent harmful bacteria from entering the liver. In another study, Li et al. (52) found that in NAFLD mice pectin could dose-dependently increase the contents of SCFAs including acetic acid and propionic acid, which may account for the beneficial effect of pectin on preventing NAFLD.

Polysaccharides could ameliorate NAFLD through modulating bile acids metabolism. Zhong et al. (53) investigated the therapeutic effect of *Ganoderma lucidum* polysaccharide peptide (GLPP) (polysaccharide-peptide ratio of 95%: 5%) on NAFLD. Results showed that GLPP increased the synthesis of bile acids through enhancing cholesterol 7-alpha-monooxygenase (CYP7A1) and 25-hydroxycholesterol 7-alpha-hydroxylase (CYP8B1) expression, while the increased bile acids promoted the farnesoid X receptor- small heterodimer partner (FXR-SHP)/fibroblast growth factor (FGF) pathway, which finally inhibited fatty acid synthesis and thus improved the steatosis in NAFLD.

**Figure 2:**
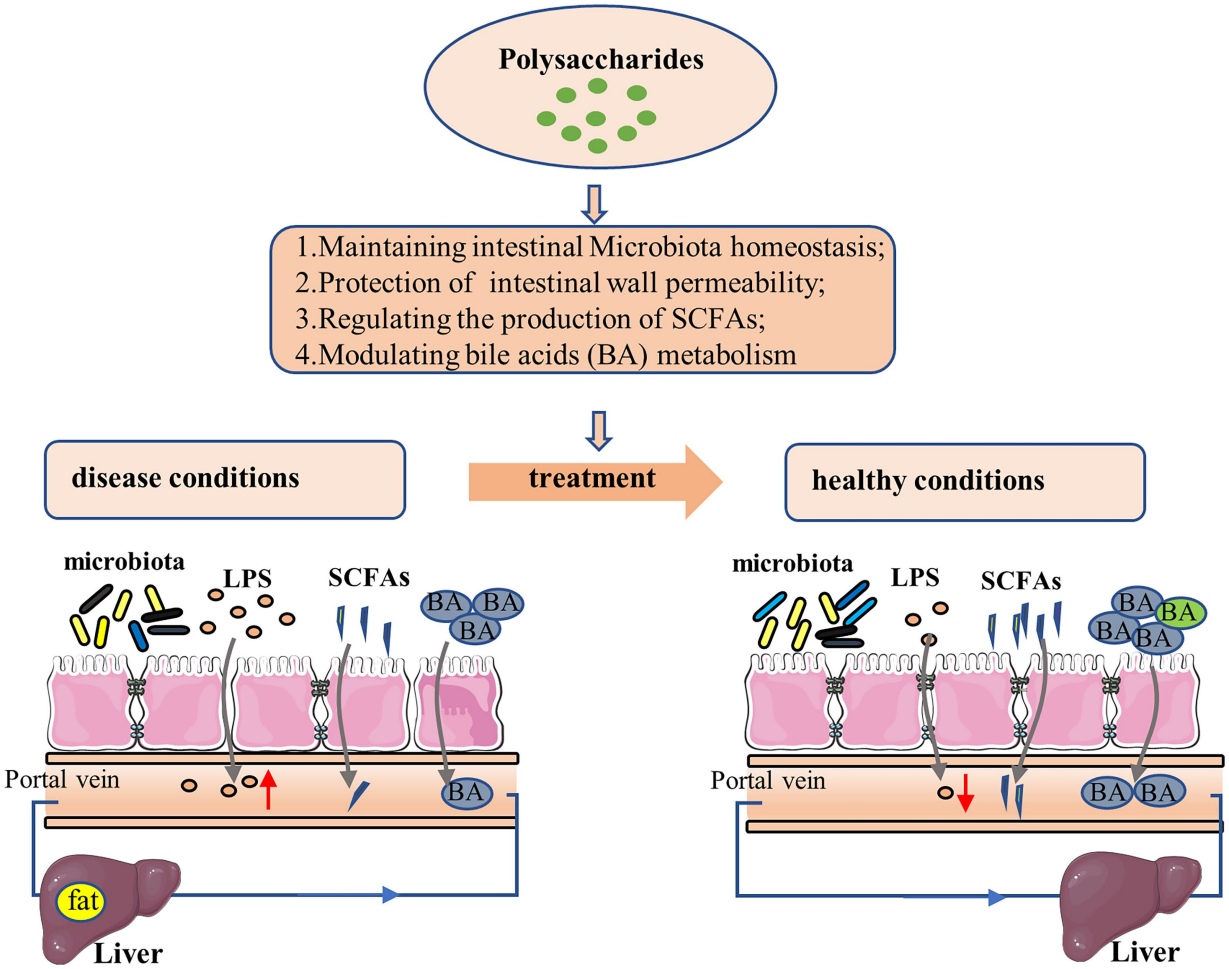
Mechanism of polysaccharide action on NAFLD *via* gut-liver axis.

From all these studies it becomes evident that polysaccharides can ameliorate NAFLD through regulating gut–liver axis at multiple interrelated levels (Figure 2). Polysaccharides could exert therapeutic effect *via* restoring intestinal microecological homeostasis. In addition, polysaccharides could reduce LPS translocation through improving intestinal barrier function, to mitigate hepatic inflammation. SCFAs, produced as by-products of polysaccharides metabolism by gut microbiota, are not only an important fuel for the body but also serve as signaling molecules involved in lipid metabolism and inflammation. Polysaccharide could regulate the relative abundance of certain SCFA-producing bacteria to affect the development of NAFLD. The metabolism of bile acid is closely associated with the homeostasis of liver, while polysaccharides could affect the metabolic homeostasis of bile acids and thereby show beneficial therapeutic effect. Therefore, polysaccharides may function as prebiotics for NAFLD treatment.

## Conclusion and prospects

Considering that the burden of end-stage liver disease will increase two- to three-fold worldwide by 2030 and NAFLD is a primary cause of end-stage liver disease and liver transplantation, slowing NAFLD progression or reversing this disease is becoming considerably imperative. Fortunately, natural polysaccharides provide a promising therapeutic alternative. Numerous preclinical trials have demonstrated that polysaccharides possess a clear and significant anti-NAFLD effect by targeting multiple mechanisms involving improvement of glucose and lipid metabolism, antioxidation, anti-inflammation, and regulation of liver-gut axis. More importantly, some polysaccharides could ameliorate NAFLD by simultaneously targeting different mechanisms with almost no side effects, thus showing intriguing potential as novel anti-NAFLD drugs or agents.

However, there are still some big challenges to face. On the one hand, polysaccharides are high-molecular weight compounds with a very complex structure, which makes quality control a big obstacle, thus overshadowing their health benefits; on the other hand, most of the polysaccharides in Table 1 are crude polysaccharides, most of which exert anti-NAFLD effect in a dose-dependent way. Apart from the actually effective components, glycoproteins, glycolipids or other structurally different polysaccharides may also be present in the mixture, which explains in part why a high-dose polysaccharides administration is usually required in preclinical studies. Of note, these impurities may also increase the potential risks or toxicity, such as hepatotoxicity or nephrotoxicity. And these adverse effects cannot be neglected in toxicological studies. Additionally, the anti-NAFLD effect of polysaccharide is closely associated with its structure. To better understand the effects of polysaccharides on NAFLD, obtaining homogeneous polysaccharides with detailed structural characteristics is particularly warranted, which is favorable to the exploration of structure–activity relationship of anti-NAFLD polysaccharides and unveil the target of anti-NAFLD action. Last but not least, the effectiveness of polysaccharide in current studies is mostly acknowledged in a preclinical stage. Therefore, systematic evaluation involving their actual efficacy and underlying mechanisms in clinic is critically essential. In the end, a myriad of effort is still needed before polysaccharides could become commercialized anti-NAFLD drugs or agents.

**Table 1:** Polysaccharides with anti-NAFLD potential reported in recent years.

Name	Source	Type	Models	Dosages	Bioactivity	Mechanism	Ref.
JFP-Ps	Jackfruit pulp	Crude	SD male rats	50, 100, and 200 mg/Kg	Regulating glucose and lipid metabolism	Increasing FFA β-oxidation (*PPARα, LPL, CPT1, HSL* ↑), and reducing *de novo* FFA synthesis (*SREBP-1c, ACC, FAS*↓)	(12)
WGHP	Walnut green husk	Crude	Kunming male mice	200, 400, and 800 mg/Kg	Regulating glucose and lipid metabolism	Improving glucose and lipid metabolism	(13)
CP	*Cichorium intybus L*.	Homogenesis	SD male rats	50, 100, and 200 mg/Kg	Regulating glucose and lipid metabolism	Increasing FFA β-oxidation (p-AMPKα, ATGL, CPT-1, and p-ACC↑), and reducing *de novo* FFA synthesis (ACC, FAS, and SCD-1↓)	(14)
			SD male rats	50 mg/Kg	Regulating glucose and lipid metabolism	Inhibiting *de novo* lipogenesis by restoring expression of genes (*Xbp1*, *Insig2*, and *Cth*); promoting FFA *β*-oxidation (*L*-palmitoylcarnitine, hexadecanoyl-CoA ↑)	(15)
			larvae zebrafish	1, 5, and 10 mg/l	Regulating glucose and lipid metabolism	Inhibiting *de novo* lipogenesis (*srebf-1*, *fas* ↓), promoting FFA *β*-oxidation (*PPARab* ↑)	(16)
ASRP	*Stropharia rugosoannulata*	Crude	Kunming male mice	400 mg/Kg	Regulating glucose and lipid metabolism	Reducing *de novo* lipogenesis (*SREBP-1c, FASN and ACC1* ↓)	(17)
GLP	*Gracilaria Lemaneiformis*	Crude	Kunming male mice	0.6, and 2.25 mg/10 g	Regulating glucose and lipid metabolism	Accelerating the conversion of cholesterol to bile acids (*LxRα* and *CYP7A1* ↑)	(18)
SFPS	*Sargassum fusiforme*	Crude	HepG2 cells; Drosophila melanogaster larvae	50 mM; 25 mg/ml	Regulating glucose and lipid metabolism	Reducing *de novo* lipogenesis (*Srebp*, *Fas* ↓), and promoting FFA *β*-oxidation (*PPARɑ*, *Cpt1* ↑)	(19)
EP	*Enteromorpha prolifera*	No mention	SD female rats	200, and 400 mg/Kg	Regulating glucose and lipid metabolism	Regulating cholesterol metabolism (*SREBP-2*, *HMGCR* ↓)	(20)
			SD female rats	200 mg/Kg	Regulating glucose and lipid metabolism	Increasing serum H_2_S level	(21)
LBP	*Lycium barbarum*	Crude	SD female rats	1 mg/Kg	Regulating glucose and lipid metabolism	Reducing *de novo* lipogenesis (*SREBP-1c*, *PPARc2* ↓), and promoting lipolysis (*ATGL*, *adiponectin* ↑)	(22)
			C57BL/6 male mice	100, and 200 mg/Kg	Regulating glucose and lipid metabolism	Reducing *de novo* lipogenesis (P-ACC ↑, FAS ↓), and promoting lipolysis (*ATGL* ↑)	(23)
GBLP	*Ginkgo biloba* leaf	Homogenesis	Male Wistar rats	100, 200, and 400 mg/Kg	regulating glucose and lipid metabolism	Attenuating insulin resistance	(24)
RHP	*Radix hedysari*	Crude	SD male rats	50, and 150 mg/Kg	Regulating glucose and lipid metabolism	Reducing lipogenesis (*SREBP-1c* ↓), and increasing FFA *β*-oxidation (*PPARα* ↑)	(25)
PCP	*Poria cocos*	Crude	C57BL/6 J male mice	0.15% PCP in HFD (w/w)	Regulating glucose and lipid metabolism	Promoting lipid transportation to blood (*Scl27a4*, *Apoc2*, *CD36* ↑), and enhancing glucose oxidative utilization	(26)
LBP	*Lycium barbarum*	Crude	SD female rats	1 mg/Kg	Antioxidation	Decreasing oxidative stress (*CYP2E1* ↓)	(29)
			LO2 cells	0.2, 0.5, and 1 mg/ml	Antioxidation	Antioxidation (SOD, CAT and GSH-Px ↑)	(30)
			LO2 cells	30, 100, and 300 μg/ml	Antioxidation	Promoting mitochondrial biogenesis (*NRF-1*, *PGC-1α* ↑)	(31)
FYGL	*Ganoderma lucidum*	Homogenesis	HepG2 cells	100, 200, and 300 μg/ml	Antioxidation	SOD and T-AOC ↑, MDA ↓	(35)
ACP	*Malpighia emarginata DC*	Crude	C57BL/6 male mice	200, 400, and 800 mg/Kg	Antioxidation	(Nrf2, HO-1, SOD, and CAT) ↑, mitigating mitochondrial dysfunction (UCP2 ↓), increasing mitochondrial complex I, IV, and V activity	(33)
SSP	*Sagittaria sagittifolia*	Crude	Male ICR mice	0.8 g/Kg	antioxidation	Nrf2, HO-1, SOD, and GSH ↑	(32)
CLPS	*Codonopsis lanceolate*	Crude	C57BL/6 male mice	100 mg/Kg	Antioxidation	Nrf2, HO-1, SOD, and CAT ↑	(34)
CPP-2	*Cyclocarya paliurus*	Crude	Female ICR mice	150, 300, and 600 mg/Kg	Antioxidation	(SOD, T-AOC, GSH-PX) ↑, MDA ↓	(36)
GP	*Gynostemma pentaphyllum*	Crude	C57BL/6 male mice	150, and 300 mg/Kg	Anti-inflammation	(*TLR1*, *TLR2,* and *TLR4*) ↓, NLRP3 ↓, and (IL-1β, IL-18rap and TNF-α) ↓	(39)
mAPS	*Astragalus mongholicus*	Crude	SD male rats	200 mg/Kg	Anti-inflammation	TNF-α, TLR4, NLRP3, and p-NF-κB ↓	(40)
Fucoidan	Marine brown algae	No mention	Male Wistar rats	100 mg/Kg	Anti-inflammation	TNF-α and IL-1β ↓	(41)
TFCP	*Tremella fuciformis*	Crude	Kunming male mice	200 mg/Kg	Anti-inflammation	IL-1β, TLR4, TNF-α, and IL-6 ↓, HNF4α ↑	(42)
CP	*Cichorium intybus L.*	Homogenesis	SD male rats	50 mg/Kg	Anti-inflammation	*Ifr1* ↓	(15)
LBP	*Lycium barbarum*	Crude	C57BL/6 N male and female mice	1 mg/Kg	Anti-inflammation	IL-6, and TNF-α ↓, NF-κB activity ↓, and NLRP3/6 ↓	(43)
			SD female rats	1 mg/Kg	Anti-inflammation	reducing NF-ĸB activity and restoring the level of IĸBa	(22)
ASRP	*Stropharia rugoso*annulata	Crude	Kunming male mice	400 mg/Kg	Anti-inflammation	(TNF-α, IL-1β, and IL-6) ↓, IL-10 ↑, (p-JNK1, p-c-Jun, and p-c-Fos) ↓	(17)
APS	Astragalus	Crude	C57BL/6 J male mice	4% APS in HFD (w/w)	Modulating gut microbiota	Modulating gut microbiota balance	(47)
MDG-1	*Ophiopogon*	Crude	C57BL/6 J male mice	0.2, 0.4, and 0.8% MDG-1 in HFD (w/w)	Modulating gut microbiota	Modulating gut microbiota balance	(48)
PCP	*Poria cocos*	Crude	C57BL/6 J mice	50, 100, and 200 mg/Kg	Modulating gut microbiota	Protecting the intestinal barrier (pyroptosis ↓), LPS translocation ↓	(49)
ASKP	*Artemisia sphaerocephala Krasch*	Crude	Kunming male mice	800 mg/Kg	Modulating gut microbiota	Protecting the intestinal barrier by increasing the relative abundance of Akkermansia	(50)
WIP	*Poria cocos*	Homogenesis	*ob*/*ob* mice	0.5, 1.0 g/Kg	Modulating gut microbiota	Regulating the production of SCFAs (butyrate ↑)	(51)
pectin	Citrus peel	Crude	C57BL/6 J male mice	4 and 8% pectin in HFD	Modulating gut microbiota	Regulating the production of SCFAs (acetic acid and propionic acid ↑)	(52)
GLPP	*Ganoderma lucidum*	Crude	*ob*/*ob* mice and ApoC3 mice	100 mg/Kg	Modulating gut microbiota	Modulating Bile acids metabolism (*CYP7A1* and *CYP8B1* ↑)	(53)

SD rats, Sprague–Dawley rats; HepG2 cells, human hepatocellular carcinoma cells.

## Author contributions

All authors listed have made a substantial, direct, and intellectual contribution to the work and approved it for publication.

## Funding

This work was financially supported by China Postdoctoral Science Fund Project (2021M690936), and Miao Pu Research Funding of Henan University of Chinese Medicine (MP2021-27 and MP2021-15).

## Conflict of interest

The authors declare that the research was conducted in the absence of any commercial or financial relationships that could be construed as a potential conflict of interest.

## Publisher’s note

All claims expressed in this article are solely those of the authors and do not necessarily represent those of their affiliated organizations, or those of the publisher, the editors and the reviewers. Any product that may be evaluated in this article, or claim that may be made by its manufacturer, is not guaranteed or endorsed by the publisher.
